# Dr. Delafield’s Address: Extract

**Published:** 1886-10

**Authors:** 


### CULLINGS FROM CONTEMPORARIES.

### HE PUT HIS FOOT IN IT.

IN the *St. Louis Weekly Review*, under the heading, "Dr. Delafield's Address," Dr. Hughes says:

At the inauguration of the Association of American Physicians and Pathologists, which occurred at Washington City last June, Dr. Francis Delafield, President, is reported as having promulgated the *raison d'etre* of the new organization in the following language: "We all like to give instruction and gain reputation, and both of these we can do in the societies already existing, but we also want a society in which we can *learn* something. And this, I take it, is the real object of the enterprise which we inaugurate to-day; to form an 'Association of Physicians and Pathologists,' to which we may come, year after year, with a well-founded hope that at each meeting we shall find something to learn."

It is sadly unfortunate that these gentlemen can only sustain the relationship of membership to the other societies to *instruct*, and must organize a new society to *learn* something.

What will the other societies do for instruction now, with these instructors and reputation makers banded together into a society of their own, and keeping the learning all among themselves? We humble learners of the other societies object.

